# Statistical methods to detect pleiotropy in human complex traits

**DOI:** 10.1098/rsob.170125

**Published:** 2017-11-01

**Authors:** Sophie Hackinger, Eleftheria Zeggini

**Affiliations:** Wellcome Trust Sanger Institute, Hinxton CB10 1SA, UK

**Keywords:** pleiotropy, statistical methods, genome-wide association study

## Abstract

In recent years pleiotropy, the phenomenon of one genetic locus influencing several traits, has become a widely researched field in human genetics. With the increasing availability of genome-wide association study summary statistics, as well as the establishment of deeply phenotyped sample collections, it is now possible to systematically assess the genetic overlap between multiple traits and diseases. In addition to increasing power to detect associated variants, multi-trait methods can also aid our understanding of how different disorders are aetiologically linked by highlighting relevant biological pathways. A plethora of available tools to perform such analyses exists, each with their own advantages and limitations. In this review, we outline some of the currently available methods to conduct multi-trait analyses. First, we briefly introduce the concept of pleiotropy and outline the current landscape of pleiotropy research in human genetics; second, we describe analytical considerations and analysis methods; finally, we discuss future directions for the field.

## 1. Introduction

The field of human complex trait genetics aims to elucidate how genetic variation affects differences in phenotypes. Most complex phenotypes are highly polygenic (i.e. they are influenced by a large number of genetic variants with moderate effects, rather than a handful of variants with large effects [1]). Since their inception in the early 2000s [2–4], genome-wide association studies (GWAS) have become the tool of choice for complex trait analysis. In the classical GWAS approach, the association of genetic variants across the entire genome with a single phenotype of interest is tested in a group of individuals. Recent years have seen a shift towards the joint analysis of related phenotypes. As a consequence of active method development in this field there are now a number of statistical tools available to detect cross-phenotype genetic associations. This review is intended to provide an overview of available methods and their relative strengths and limitations.

### 1.1. Types of pleiotropy

**Figure 1.**
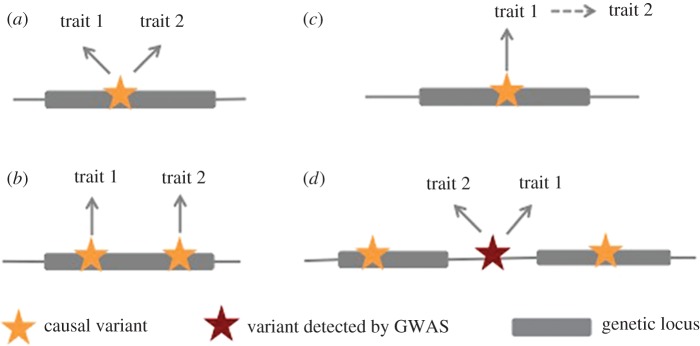
Schematic representation of different scenarios for cross-phenotype associations. Such effects might arise due to biological pleiotropy, whereby causal variants for two traits colocalize in the same locus (*a*,*b*), due to mediated pleiotropy, whereby a variant exerts an effect on one trait through another one (*c*), or due to spurious pleiotropy, whereby causal variants for two traits fall into distinct loci but are in LD with a variant associated with both traits (*d*).

The term ‘pleiotropy’ was coined over 100 years ago by German scientist Ludwig Plate to describe the phenomenon of a hereditary unit affecting more than one trait of an organism [5]. Since then, pleiotropy has been a topic of extensive research and debate. Before human genetics began to gain traction, pleiotropy was mainly studied in model organisms and, on a more theoretical level, in evolutionary biology [5,6]. Over the course of the past decades there have been several proposals on how to classify different types of pleiotropy [5,7–9]. With regards to GWAS, it is important to note that cross-phenotype associations can arise due to several reasons, not all of which are biologically meaningful [7,8]. Solovieff and colleagues [8] described three broad categories of pleiotropy in the context of complex traits:

In the case of biological pleiotropy, causal variants of different traits fall into the same gene or regulatory unit (e.g. transcription factor binding sites) [8]. In GWAS this could manifest itself in the form of two different variants in the same region tagging the same or two separate causal variants, or as one variant tagging the causal one (figure 1*a*,*b*). In practice, fine-mapping and molecular studies are required to confidently distinguish between these different scenarios [8].

Mediated pleiotropy refers to the case where a variant directly affects one trait, which in turn affects another (figure 1*c*). GWAS will still pick up an association of the variant with the second trait, but this association will disappear when conditioned on the first. Causal inference can be achieved through Mendelian randomization studies, which have been widely used in genetic epidemiology [8,10–12]. An example is the association of the *FTO* gene with osteoarthritis (OA) [13], which was shown to exert its effect on OA through body mass index (BMI) [14].

Finally, cross-phenotype associations can also arise due to spurious pleiotropy. At the planning stage of a study, design artefacts may lead to inaccurate results. For example, ascertainment bias or misclassification of cases can both inflate genetic overlap estimates. At the analysis stage, causal variants in different genes may be tagged by the same GWAS variant (figure 1*d*). A classic example of this is the human leucocyte antigen (HLA) region on chromosome 6. Due to its high gene density and extensive linkage disequilibrium (LD), GWAS signals within the HLA region are difficult to map finely. While the HLA locus has been associated with a range of diseases [13,15–20], most prominently immune-mediated ones, it remains unclear to what extent these disorders share the same causal risk variants or genes.

### 1.2. Pleiotropy in human complex traits

In 2011, a systematic evaluation of associations reported in the NIHGR GWAS catalogue found that 4.6% of variants were associated with more than one trait [21]. This number is likely to have grown, as GWAS signals have been continuously added to the database.

Many cross-phenotype effects are not surprising. For example, variants in the *DSP* gene are associated with chronic obstructive pulmonary disease, as well as pulmonary fibrosis and lung function traits [22]. Others are perhaps less intuitive and can shed light into hitherto unknown connections between traits. For example, variants in the *ASTN2* gene have been shown to affect both risk to osteoarthritis [13] and migraine [23,24]. These seemingly unrelated diseases might share pathways involved in pain perception.

Until a few years ago, the focus of many consortial efforts was to combine datasets of one phenotype for large-scale GWAS and meta-analyses [15,25,26]. For many traits, results from these studies are now publicly available, providing an excellent resource for cross-phenotype analyses using summary statistics. As the appreciation of pleiotropic effects has gained traction in the scientific community, cross-disorder analyses of several related traits have been increasingly carried out to disentangle shared and disease-specific genetic determinants [27–30].

The establishment of genome-wide genotyped biobanks [31] and cohorts with in-depth phenotype information [32] has also made it possible to perform multi-trait analyses on the same sample set [12,33], for example through phenome-wide association studies (PheWAS), where the association of each genetic variant with all phenotypes in a dataset is tested [34–37].

One challenge of the PheWAS approach is the high multiple-testing burden that grows as the number of traits and variants tested increases [9]. Although this can be partly circumvented by performing targeted PheWAS at a selected number of variants hypothesized to exert pleiotropic effects [9,38], other challenges such as consistent phenotyping and selection of appropriate covariates remain [34].

Investigating pleiotropy in human traits not only holds the potential to uncover additional associations, but could additionally help to redefine disease classifications. This is of particular interest in disorders for which the aetiopathology is unclear, and for which current diagnostic tools might be inadequate. For example, psychiatric conditions are highly comorbid, and until recently [15,39] have been mostly refractory to GWAS [40]. Comparisons of different psychiatric disorders have shown that the genetic overlap among them is extensive [27,41], and that certain pairs of diseases are genetically more similar than others [41]. Together these findings suggest that shared biological mechanisms cross diagnostic boundaries, and might aid the development of more accurate disease classification systems.

## 2. Analytical approaches

### 2.1. Study design considerations

There are some practical considerations to be taken into account when selecting an appropriate method for multi-trait analysis.

First, the type of data available will determine which statistical approach is applicable. Due to limitations of data sharing policies it might not be possible to obtain individual-level genotype data for all traits analysed.

Second, the type and number of traits to include must be considered: some approaches require all traits to be continuous, while others also allow for dichotomous traits or a combination of both. Several methods, such as colocalization tests [42,43] or genetic correlation analyses [44], can currently only accommodate two traits at a time, while others lose power with an increasing number of traits [45].

Finally, if each trait is measured on a different set of individuals, sample overlap between each dataset will need to be accounted for. This has been implemented in several methods [43,44,46]. Ideally, the exact number of overlapping individuals will need to be accounted for. However, this is often not possible when using data from publicly available GWAS. One way to estimate the extent of overlap is to calculate the Pearson's correlation of the *Z*-scores of all independent, non-associated variants from two studies [43], although other methods have also been proposed [47–49].

### 2.2. Overview of methods

Pleiotropy analyses can be broadly classified into three categories according to the level at which they assess genetic overlap: genome-wide, regional and single variant.

Genome-wide methods are currently only available for pairwise trait comparisons, and can be used as an initial assessment of the global genetic overlap between two traits.

The latter two approaches aim to detect cross-phenotype effects at distinct genomic regions and at a single variant, respectively.

Region-based methods bin variants into groups based on pre-defined criteria, such as LD-blocks or gene boundaries, and then test for cross-phenotype effects within each group. An advantage of such approaches is that they alleviate the multiple testing penalty incurred by single-point analyses; furthermore, they can increase power by combining information across biologically meaningful units.

Since variant-level methods test each variant separately, they provide the highest resolution. On the other hand, they are less powerful in situations where each trait is associated with a different variant in the same functional unit, and might fail to identify these cross-phenotype effects unless all relevant variants are in at least moderate LD.

The above analysis approaches can be further sub-divided based on their underlying statistical framework into univariate and multivariate. Univariate methods combine summary statistics of single-trait GWAS to search for cross-phenotype effects. This means that analyses can be carried out with each trait measured on a distinct set of individuals. Multivariate methods, on the other hand, jointly model all traits in a statistical framework, which requires that all individuals included in the study have phenotype information for all traits analysed. The statistical difference between uni- and multivariate methods is best illustrated by the example of linear regression analysis: for univariate regression, the response variable (i.e. the phenotype) will be a vector, with one data point for each individual in the study; for multivariate regression, the response variable will be a matrix, where each row represents an individual and each column represents one phenotype. Although there are exceptions, these categories are often analogous to distinguishing between methods requiring only summary data and individual-level information, respectively.

### 2.3. Genome-wide methods

Polygenic risk scores (PRS; or genetic risk scores) were initially used in genetic epidemiology to test how well a set of variables could predict, or distinguish between, case-control status in a study sample [50–53]. In the context of GWAS, the risk variables comprise variants known to be associated with a given trait. Odds ratios (ORs) for these variants from a ‘base’ GWAS are then used to construct scores for each individual in an independent ‘target’ dataset. Using logistic (binary trait) or linear (continuous trait) regression to relate phenotype and score, the proportion of phenotypic variance explained in the target data by the base risk variants can be directly estimated.

This framework can also be applied to two different traits [27,36,54,55]. Using this approach, Purcell and colleagues [54] showed that risk scores for bipolar disorder are significantly associated with schizophrenia, and that the variance in phenotype captured by the risk variants could be increased by relaxing the *p*-value threshold for variant inclusion (rather than using only genome-wide significant variants). One reason for this could be that many variants with a true effect on the phenotype did not reach genome-wide significance in the base study. This is especially likely for highly polygenic traits, for which only a fraction of the heritability can be explained by currently known risk variants.

Genetic correlation (rg) captures the extent to which genetic factors influence the covariance of two traits. Multivariate methods for genetic correlation analysis include GCTA [28,56], BOLT-REML [57] and mvLMM [58]. GCTA and BOLT-REML use restricted maximum-likelihood estimation to compute rg between two traits of any type (i.e. two binary, two continuous, or one binary and one continuous), while the mvLMM algorithm is more similar to GEMMA and can only accommodate normally distributed traits. Individual-level genotype data are required as input, and samples need to have phenotype values for both traits analysed. For disease traits, overlap between the cases and controls for each trait should be negligible. While all three methods use similar algorithms, BOLT-REML and mvLMM are more efficient than GCTA in terms of run time and memory usage [57,58].

More recently, a univariate method for genetic correlation analysis, cross-trait LD score regression (LDSC), requiring only summary statistics, has been developed [44,59]. Like its multivariate counterparts, LDSC can handle any combination of traits and can adjust for sample overlap. The method requires the use of a reference panel for LD estimation. This is of particular importance when analysing distinct GWAS performed on populations of different ancestries. The LD Hub database, which acts as both a central aggregation of public summary statistics and an online interface for LDSC, enables systematic comparisons between a range of traits [60]. As the authors of LDSC point out, it is important to distinguish genetic correlation from pleiotropy [44]. A near-zero estimate of genetic correlation between two traits does not necessarily mean that they share no common risk loci. For example, there could be no directionality to their genetic relationship (i.e. at some shared loci the risk allele is the same for both traits, while at others the risk allele for one trait is protective of the other). An example of the latter scenario is the rs7501939 variant in *TCF2*, for which the C allele confers increased risk for prostate cancer and decreased risk for type 2 diabetes [61]. As for PRS, if either or both of the input datasets are underpowered, this could also lead to a falsely low estimate of r_g_. Conversely, in the case of disease traits, genetic correlation could be inflated due to ascertainment bias or misclassification of cases [8].

### 2.4. Regional methods

The pleiotropic region identification method (PRIMe) [62] defines regions by designating the most strongly associated variant among the included traits as a ‘driver’, and all variants in LD with it as ‘passengers’. The next most strongly associated variant is then considered: if it has been already assigned as a passenger, it is skipped; otherwise it is designated as a driver, and so on. This process is repeated iteratively until the genome is divided into non-overlapping blocks consisting of one driver and zero or several passengers. The pleiotropy index of each region is defined as the number of traits with association *p*-values below some threshold in that region. The statistical significance of the index can be calculated using two separate approaches for traits measured on overlapping or non-overlapping datasets, respectively.

In 2013 Giambartolomei and colleagues described a Bayesian colocalization model to identify genomic regions of colocalizing expression quantitative trait loci (eQTL) and GWAS signals [42]. This method was then extended to account for sample overlap, and implemented in a software package (gwas-pw) to enable simplicity of use for the pairwise comparison of GWAS summary statistics [43]. The model integrates the effects of all variants in a pre-defined region, such as approximately independent LD blocks [63]. It generates posterior probabilities for each of five hypotheses, the two most relevant being that in a given region the traits share one causal variant, and that they each have a separate causal variant. An advantage of this approach over many variant-level methods is that it evaluates the evidence for *both* traits being associated with a given region, thus making it possible to distinguish from the scenario of one trait driving an observed signal.

Multivariate methods for locus-based analysis also exist: Tang & Ferreira [64] extended a previously developed method using canonical correlation analysis (CCA; see §2.6) to perform association analysis between two traits and sets of genetic markers, for example within genes. Further development of the method also allowed for multiple gene-multiple phenotype tests [65]. A recently published method, metaCCA, performs CCA on summary data from single-trait GWAS at both a variant- and locus-level (see §2.5) [66]. The use of functional linear models for the analysis of multiple variants and multiple traits has also been proposed [67]. A limitation of this and of CCA-based approaches is that they are not applicable to disease or non-normally distributed traits. This was overcome by Lutz and colleagues [68] by using a permutation-based approach that accommodates any combination of traits (see §2.6). Finally, mtSET uses a multivariate mixed model with two variance components to account both for inter-individual variation (e.g. due to population structure or relatedness) and for variation among the variants being tested together [69].

#### 2.4.1. Rare variant tests

The substantial drop in sequencing costs over the past decade together with the establishment of better reference panels for imputation have made association studies of low frequency and rare variants feasible [70,71]. Methods for rare-variant studies usually group several variants together and perform an association test with this composite genotype. They are generally more powerful than testing individual rare variants [72], and have been the tool of choice for single-trait studies [73]. Two of the most popular burden test methods are kernel-based tests (such as SKAT [74]) and collapsing tests [75]. These and other approaches have been reviewed in detail elsewhere [73].

While some of the multi-trait methods described above are applicable to both common and low-frequency markers [67–69], approaches have also been specifically designed for rare variants. The methods described below all rely on individual-level data with phenotypes measured in the same set of individuals.

Wu and Pankow extended univariate SKAT for the application to multiple continuous traits [76]. Another method, MAAUSS, also builds on the SKAT algorithm, including a variance-covariance matrix that allows for the joint modelling of multiple phenotypes [77]. Multiple binary or a mixture of binary and continuous traits can be analysed by MAAUSS through integration of the generalized estimating equation framework (see also §2.6).

In adaptive weighting reverse regression (AWRR) [78], the genotypes in a set of variants are first combined, weighted by the strength of association and direction of effect of each variant; the resulting variable is then regressed on multiple traits and a score test used to assess significance. This reverse regression approach is similar to other methods discussed here (see §2.6), and can incorporate large numbers of traits of any kind.

### 2.5. Single-point univariate methods

With the increasing availability of summary data from large-scale GWAS, an important question has been how to harness these data to perform pleiotropy analyses. Perhaps the simplest way to search for cross-phenotype effects is to decide on a *p*-value threshold and declare all variants that fall below this threshold for a group of traits as cross-phenotype associations [8]. However, this approach can be underpowered, as even with large sample sizes truly associated variants with sub-threshold *p*-values will be missed. Consequently, a number of methods to statistically combine summary data for multiple traits have been developed (table 1).

**Table 1.** Univariate methods for single-point association analysis and variant prioritization. impl., implementation.

method	ref.	PMID	year	data	*n* traits	trait type	impl.
CPMA	[79]	21852963	2011	*p*-values	>2	any	R
ASSET	[45]	22560090	2012	betas, SEs	≥2	any	R
CPASSOC	[80]	25500260	2015	*Z*-scores	≥2	any	R
MultiMeta	[54]	25908790	2015	betas, SEs	≥2	any	R
MTAG	[81]	NA	2017	betas, SEs	≥2	any	Python
cFDR	[82]	25658688	2015	*p*-values	2	any	R
Bayesian overlap	[83]	26411566	2015	*p*-values	2	any	NA
metaCCA	[66]	27153689	2016	betas, SEs	≥2	any	R
GPA	[84]	25393678	2014	*p*-values	2	any	R
GPA-MDS	[85]	27868058	2016	*p*-values	≥2	any	R
fastPAINTOR	[86]	27663501	2017	*Z*-scores	≥2	any	C++
EPS	[87]	27153687	2016	*p*-values	2	any	Matlab
RiVIERA-MT	[88]	NA	2016	*p*-values, betas, SEs	≥2	any	R

#### 2.5.1. Extensions to meta-analysis

In the classical meta-analysis approach, *p*-values or effect sizes are combined across multiple studies of the same trait [89]. For the latter, effects are typically either assumed to be consistent across studies (fixed effects meta-analysis) or allowed to vary (random effects meta-analysis). However, a genetic variant might have the opposite effect on two traits. While this can be circumvented by applying a directionality-agnostic *p*-value-based meta-analysis, there are some limitations, such as the inability to obtain an overall effect estimate [89]. Therefore, these standard approaches are best-suited to groups of traits/disorders assumed to have similar underlying biological mechanisms [27]. The meta-analysis framework has been adapted to accommodate this and other issues that arise when combining several different traits.

Cotsapas and colleagues developed a cross-phenotype meta-analysis (CPMA) method that tests for the presence of two or more trait associations at a variant [79]. This has the advantage of protecting against the scenario of one trait driving the association. CPMA only requires *p*-values as input and is thus robust to heterogeneous effect directions. Since CPMA compares the distribution of *p*-values for all traits at a variant to the null hypothesis of uniformity, it is well suited for moderate to large numbers of phenotypes, but less so for pairs of traits.

In a generalization of fixed-effects meta-analysis, all possible subsets of traits are evaluated to identify the one with the maximum absolute *Z*-statistic at a variant [45]. The approach, termed ASSET, takes effect estimates as input, and can also be used to identify disease subtypes within case-control data. Extensions were also proposed to account for sample overlap and effect heterogeneity between traits [45]. The method expects all traits to be of the same type, and the number of tests performed grows exponentially with the number of traits, decreasing power. Using ASSET, investigators have identified three loci associated with five autoimmune disorders, as well as risk loci associated with different cancers [90].

Zhu and colleagues developed two meta-analysis test statistics to detect cross-phenotype associations assuming homogeneous and heterogeneous effects across studies, respectively [80]. The tests are implemented in the R package CPASSOC, and work with both univariate (i.e. one trait per cohort) and multivariate summary statistics (i.e. several traits measured in each cohort). CPASSOC requires the specification of an inter-cohort correlation matrix. Since the true phenotypic correlation is unknown in the absence of raw data, this can be derived from summary statistics and—similarly to approaches outlined above—accounts for overlapping samples. Applying CPASSOC to anthropometric trait summary data from the GIANT consortium identified one novel genome-wide significant locus within the *TOX* gene missed by conventional meta-analysis [91].

A recently published method, MultiMeta, enables the joint analysis of summary statistics obtained from multivariate GWAS, such as multivariate LMM analysis (see §2.6) [92]. MultiMeta generalizes single-trait inverse variance weighted meta-analysis to allow each variant to have a vector of effect estimates (one for each trait included).

The Multi-Trait Analysis of GWAS (MTAG) tool takes a slightly different approach to the above-mentioned methods: in a generalization of inverse variance weighted meta-analysis, it incorporates effect estimates from multiple traits and outputs adjusted effect estimates for *each* trait separately [81]. Sample overlap is accounted for by LD score regression. If there is no genetic correlation between any of the traits, and each trait has been measured on an independent sample, the MTAG effect estimates are equivalent to the single-trait estimates.

#### 2.5.2. Bayesian methods

Conditional false discovery rate (cFDR) can detect variants associated with one ‘principal’ trait, given its *p*-values of association with both the ‘principal’ and the ‘conditional’ trait fall below a certain *p*-value threshold [82]. An extension to allow for shared controls has also been developed [46]. Conditional FDR can be used as a variant prioritization tool, to detect additional associated variants with one trait by leveraging information on their association with a second trait; furthermore, the method can serve to detect cross-phenotype effects explicitly by taking the maximum of the cFDRs computed for each of two traits [93].

Asimit and colleagues developed a Bayesian approach that adjusts for sample size (and, consequently, power) differences between studies [83]. Based on *p*-values from two input studies, the method tests for an excess of shared signals and can be used to identify a list of variants with evidence of association for both traits.

#### 2.5.3. Other approaches

In metaCCA, a method based on canonical correlation analysis (CCA), single-trait summary statistics together with genotype data from a reference panel are used to reconstruct the genotype–phenotype covariance matrix for each study [66]. Weighted averages of these matrices are then combined across studies, and can be used to perform a multi-trait meta-analysis at a single variant or a genetic locus (based on LD).

#### 2.5.4. Variant prioritization and fine-mapping

In order to aid variant prioritization and fine-mapping, the integration of GWAS data and functional annotations (such as chromatin marks) as well as transcriptomic data has become popular [94–96]. Some methods now allow for the inclusion of multiple GWAS of one or several traits.

The first tool allowing for the simultaneous modelling of multiple GWAS and annotation data was GPA [84]. The model tests for enrichment of annotations of (multi-)trait associated variants in functional datasets (e.g. eQTL or histone modifications). Although it can be easily extended to more than three traits/GWAS datasets, the degrees of freedom of the resulting chi-squared test grow with every trait tested, leading to a loss of power. As only binary annotations can be included in this model, the investigators built a revised approach, GPA-MDS, which combines multidimensional scaling with the GPA algorithm to draw visual representations of the relationships between multiple phenotypes [85].

FastPAINTOR, an extension to the single-trait fine-mapping algorithm PAINTOR [97], additionally models the LD structure at a locus which can improve accuracy [86]. Following a similar model to GPA, a Bayesian method entitled EPS also accounts for LD, but infers association at the gene- rather than the variant-level, and can incorporate gene expression data from a large number of tissues [87]. RiVIERA-MT further allows for multiple causal variants within one locus [88].

### 2.6. Single-point multivariate methods

As the availability of large-scale genetic datasets with multiple phenotype measurements increases, the focus of method development for multi-trait analyses has shifted towards multivariate methods that use individual-level data rather than summary statistics (table 2).

**Table 2.** Multivariate methods for single-point association analysis. impl., implementation; ND, normally distributed.

method	ref.	PMID	year	data	*n* traits	trait type	impl.
FBAT-PC	[98]	16646795	2004	raw	≥2	any	C
PCHAT	[99]	17922480	2008	raw	≥2	any	Fortran
AvPC	[100]	27876822	2016	raw	≥2	any	NA
mvPlink	[101]	19019849	2009	raw	≥2	any	C++
MTMM	[102]	22902788	2012	raw	2	ND	R
GEMMA	[103]	24531419	2014	raw	≥2	ND	C/C++
mvLMM	[58]	25724382	2015	raw	≥2	ND	Python
GAMMA	[104]	27770036	2016	raw	≥2	ND	R
B_EGEE	[105]	18924135	2009	raw	2	any	Fortran
PleioGRiP	[106]	23419378	2013	raw	2	binary	Java
mvBIMBAM	[107]	23861737	2013	raw	≥2	ND	C/C++
Kendall's tau	[108]	20711441	2010	raw	≥2	any	NA
MultiPhen	[109]	22567092	2012	raw	≥2	any	R
ATeMP	[110]	26479245	2015	raw	≥2	any	NA
BAMP	[111]	26493781	2015	raw	≥2	any	NA
TATES	[112]	23359524	2013	*p*-values	≥2	any	R/Fortran
extension to O'Briens	[113]	20583287	2010	raw	≥2	any	upon request
Trinculo	[114]	26873930	2016	raw	≥2	categorical	C
log-linear model	[115]	21849790	2011	raw	≥2	binary	NA
PET	[116]	25044106	2014	raw	2	ND	R
PLeiotropySNP	[68]	27900789	2016	raw	≥2	any	R

These approaches are generally more powerful than combining test statistics from univariate GWAS, as the inter-trait covariance can be accounted for [117,118]. In this section we will outline some of the methods available to conduct cross-phenotype analyses with individual-level data. Unless otherwise stated, all methods described in this section are based on multivariate models. For a more detailed discussion of the statistical properties of different multivariate approaches we refer the reader to two excellent reviews [117,119].

#### 2.6.1. Dimension reduction

One efficient way to deal with multivariate phenotypes is to first apply a dimension reduction technique that collapses the individual trait values, and then perform an association between genotype and this new set of variables. Principal component analysis (PCA) derives linear combinations of the phenotypes that explain the greatest possible covariance between them [117]. This approach was first used in linkage studies with multiple trait measurements, where principal components (PCs) were calculated to maximize the heritability in the data [120]. Extensions of this method were developed for family [98] and population-based studies [99]. The latter method, called PCHAT, requires splitting of the study sample into a ‘training set’ to derive the PCs and an ‘analysis set’ to perform the association, which can lead to a loss of power.

Recently, it has been shown that PCs can also be combined across individual multi-trait studies for meta-analysis. Average PCs are derived from the weighted means of the loadings (i.e. the linear combination of traits) in each study [100].

In contrast to PCA, CCA derives linear combinations of the traits that explain the greatest amount of covariance between a given variant and the traits. The first implementation of CCA for multivariate phenotypes was developed by Ferreira & Purcell [101], and later extended for gene-based analyses [64,65] (see §2.4).

#### 2.6.2. Multivariate mixed models

Linear mixed models (LMMs) are an extension of standard regression analysis incorporating both fixed and random effects, and have gained popularity in GWAS due to their ability to handle relatedness among individuals [121,122]. Multivariate LMMs can be used for association testing with multiple phenotypes. They model association between a genetic marker and the traits as the fixed effect, and the inter-trait covariance as the random effect [117]. While multivariate mixed models are generally more powerful than standard univariate association tests, they perform les well when the traits under consideration are only weakly correlated [102], and assume phenotypes to be normally distributed, which does not allow for the inclusion of disease traits.

Korte and colleagues first applied multivariate LMMs to pairwise quantitative trait measurements in a human cohort [102]. The method, MTMM, showed increased power to detect loci compared with single-trait LMMs, and can also be used to decompose overall trait covariance into genetic and environmental factors.

Fitting multivariate LMMs requires a computationally intensive parameter estimation step, which until recently impeded their application to more than two traits [58,102,103]. A multivariate extension of the GEMMA algorithm [103] can accommodate a moderate number of phenotypes (between 2 and 10) and also shows substantially faster computation times compared with MTMM. A further improvement was achieved by Furlotte & Eskin with their matrix-variate linear mixed model (mvLMM), with runtime scaling linearly, rather than cubically, with the number of samples included [58]. The same research group also developed GAMMA, a mixed model that accurately adjusts for population structure, with computation time scaling linearly with the number of phenotypes, enabling it to jointly analyse large numbers of traits (more than 100) [104].

#### 2.6.3. Generalized estimating equations

Generalized estimating equations (GEEs) are a multivariate method to jointly analyse non-normally distributed phenotypes [119,123]. As the standard GEE approach requires traits to have the same underlying distribution, combining binary and continuous traits is not straightforward. B_EGEE was developed to overcome this constraint, using a regression procedure to incorporate two models with different underlying link functions into a unified equation system [105]. This method is implemented for two traits, although the authors suggest that the incorporation of multiple traits be possible [105]. While not as powerful as LMMs, GEEs have been used in family-based tests for both single- and multi-trait analysis due to their ability to model the random effects of relatedness [124,125].

#### 2.6.4. Bayesian methods

Bayesian statistics allow for a model comparison between several alternative hypotheses, making them an attractive tool for pleiotropy analysis [42,43,107,126]. PleioGRiP is based on naive Bayesian classifiers that can be used to both test for association of a variant with two or more phenotypes and to perform genetic risk prediction [106,126].

A model-selection framework proposed by Stephens returns Bayes factors for each possible partitioning of phenotypes into one of three categories: unassociated, directly associated, or indirectly associated with a genetic marker [107]. The weighted average of these Bayes factors gives the overall evidence that any phenotype be associated. At markers where the evidence against this global null is strong, the individual Bayes factors can be used to determine which traits are likely to drive the association. The framework is implemented in the software mvBIMBAM and has been used to identify variants associated with low- and intermediate-density lipoprotein subfractions [127]. While it has the advantage of accepting either individual-level or summary data as input, it is not applicable to non-normally distributed traits.

A Bayesian multivariate regression framework was also implemented in the widely used GWAS software SNPTEST [128].

#### 2.6.5. Other approaches

A non-parametric score test based on Kendall's tau makes no assumption about the distribution of traits being analysed, and can thus incorporate any type of trait [108]. A limitation is the inability to adjust for covariates.

Another way to allow for the inclusion of traits with mixed distribution is to reverse the regression of standard GWAS. MultiPhen performs ordinal regression of the genotype (number of minor alleles at a marker) on multiple phenotypes and tests for association using a likelihood ratio test [109]. Alternatively, a score statistic can be used to test for association, which is computationally more efficient and equivalent to Kendall's tau. To prevent loss of power for non-normally distributed phenotypes, ATeMP first standardizes traits using a normalized rank or ordinal residual transformation [110]. Alternatively, allele-specific information can be related to trait values in binomial regression [111]. The latter approach, termed BAMP, is slightly more powerful in the case of non-normally distributed phenotypes [111,129].

Trait-based Association Test that uses Extended Simes procedure (TATES) is a model-free approach that combines univariate *p*-values of different traits while taking into account the correlation between the traits [112]. Even though the test is based on summary statistics, individual-level data need to be available to calculate the correlation matrix. All samples are expected to be phenotyped across all traits.

Based on a univariate method developed by O'Brien [113], which derives a weighted sum of univariate test statistics, Yang and colleagues proposed two approaches that allow for effect heterogeneity among studies [130]: a sample-splitting approach, similar to that used in PCHAT [99], and a cross-validation approach. An advantage of this framework is that it can deal with missing phenotype data well (i.e. not all traits being measured on the same set of samples).

Multinomial models are a generalization of logistic regression, where the outcome variable (in this context the phenotype) can take more than two possible outcomes [131]. Such models are applicable to disease traits where cases can be classified into two or more sub-classes, for which risk variants might exhibit heterogeneous effects. While not technically multivariate, this type of analysis requires individual-level data. Bayesian and frequentist multinomial regression has been implemented in the software package Trinculo [114]. The Bayesian approach was recently used to determine which of a group of six immune disorders was associated with previously identified risk loci [132].

#### 2.6.6. Explicit tests of pleiotropy

Model selection procedures are effective tools to discern associated from non-associated traits. A log-linear framework proposed by Lee and colleagues [115] distinguishes between different causal models of association, and can identify effects specific to a subgroup of individuals using information criteria. The method is only applicable to disease traits, and has been used to identify subtype-specific effects in migraine [133] and schizophrenia [134], as well as cross-disorder associations in psychiatric diseases [28,135]. Information criteria can also be used in an ordinal regression framework, such as MultiPhen [109], to identify the group of traits driving an association [129].

The pleiotropy estimation and test (PET) tool determines the pleiotropic effect of a variant on two continuous traits as the proportion of inter-trait correlation explained [116]. This quantity is assessed against the null hypothesis that neither trait is influenced by the variant.

Lutz and colleagues extended the above method for non-continuous traits and for more than two phenotypes, as well as for rare variant or region-based tests (see also §2.4) [68]. The approach is based on performing standard univariate association analyses for each phenotype, followed by permutations of the phenotypes; evidence for pleiotropy is assessed by two alternative ways each of which compare the univariate *p*-values to the permutation-derived ones. The method tests for evidence against the null hypothesis of no association with *all* included phenotypes, and thus excludes the possibility of a signal being driven by one trait only. Therefore, if all but one of the analysed traits are associated with a variant or gene, the method will not reject the null.

### 2.7. Single-point methods summary

Several comparisons of different multi-trait methods have been conducted to date, testing power and type I error rates, as well as computational performance under different scenarios [118,129,136–138]. Since each report focused on a different combination of methods, the emerging picture seems to be that the most suitable method depends on individual study set-up.

Multivariate approaches are generally more powerful than univariate methods [138], unless only one trait is associated with a genetic marker or all traits are very highly correlated [102]. It is therefore advisable to perform both multivariate and univariate association tests in a complementary way [103]. This will not only enable the detection of additional signals, but also aid the interpretation of a multivariate association (i.e. which trait(s) is/are driving the signal). Since only a handful of currently available methods explicitly test for cross-phenotype effects, considering univariate association statistics also guards against false positive multi-trait associations.

When combining summary statistics across multiple traits in a univariate fashion, an important consideration is the power of individual studies. As for regional or genome-wide methods, single-point methods will fail to detect cross-phenotype associations if the input datasets are underpowered. Another important aspect is the ancestry of input study samples, especially for methods requiring the specification of reference panels [66], for which combining studies from different populations might lead to spurious results.

The implementation and computational efficiency of different methods are additional important considerations. This is especially relevant to multivariate methods using individual-level genotype data, which generally require more memory and processing power. Most of the multivariate approaches outlined above have been tested on small to moderate sample sizes (*n* < 5000), making it hard to predict their performance on large datasets. For example, methods implemented in the programming language R will generally be less memory-efficient than programs implemented in C or C++.

### 2.8. Detecting mediated pleiotropy

**Figure 2.**
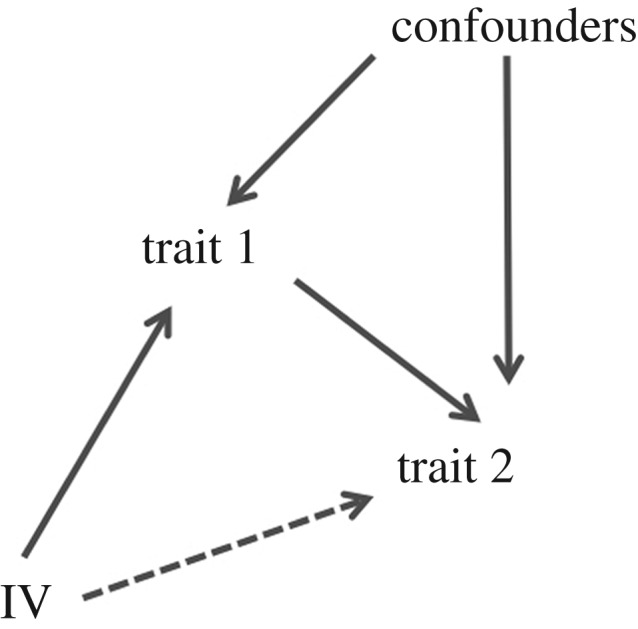
Directed acyclic graph of the Mendelian randomization model. IV, instrumental variable.

Determining whether the correlation between two traits is due to a causal link (i.e. trait 1 is a causal risk factor for trait 2) or due to confounding factors such as environmental exposures can be achieved through Mendelian randomization (MR). Notably, while most methods outlined in this review aim to detect biological pleiotropy and are confounded by mediated pleiotropy, the opposite is true for MR. MR uses information on the association of one or several genetic markers—instrumental variables (IVs)—with each trait to infer whether or not trait 1 causally influences trait 2 [11,139–142] (figure 2). An early example of MR is a study published in 2005 which concluded that, contrary to prior belief, C-reactive protein levels were not causal for metabolic syndrome [11]. If both traits were measured on the same samples an MR can be performed via two-sided least-squares analysis, where trait 1 is first regressed onto the IVs, and trait 2 is then regressed on the predicted values of trait 1 from the first regression. The effect size derived from the second regression is the MR estimate. If only summary data are available, or the associations for each trait were derived from different studies (referred to as two-sample MR), the Wald estimator can be used instead: this is the ratio of the effect of the variant on trait 1 over its effect on trait 2 [143].

In order to be a valid IV, three key assumptions about the genetic marker must be met: first, the marker is associated with trait 1; second, the marker is not associated with any confounding variables, such as environmental exposures; and third, the marker is not associated with trait 2 when conditioning on trait 1. The first two assumptions are usually easy to fulfil in a GWAS context. The first assumption also implies that the function of the gene or marker used as an IV is known *a priori*. Consequently, MR is not a method to detect new genotype–phenotype associations [141]. Some consideration should be given to assumption 2, which can be violated in the case of population stratification [141,144]. Arguably the biggest uncertainty is the third assumption, which will not hold if the variant or variants used independently affect both trait 1 and trait 2 (i.e. if the variants are truly pleiotropic).

To overcome this limitation, several approaches have been proposed to both detect and subsequently adjust for pleiotropy in MR settings [145–147]. The inclusion of multiple independent variants is a prerequisite for such analyses: if all of the variants satisfy the IV assumptions there should be no heterogeneity between their individual MR estimates [141,145]. In other words, in the case of no pleiotropy, MR estimates of each variant will only vary by chance.

If individual-level data are available and both traits have been measured on the same sample, the Sargan test can be used to assess evidence against the null of all MR estimates being the same [144]. For two-sample MR, an adaptation of inverse-variance weighted meta-analysis can be used to combine Wald estimators across several variants [148]. This approach assumes that the three IV assumptions hold. The Cochran Q-statistic and the related *I*^2^ index can be used to test for heterogeneity between individual IV estimates [145]. In Egger regression, variant-trait 2 effect sizes are regressed on the variant-trait 1 effect sizes with an unconstrained intercept [147]. An intercept term significantly different from zero is indicative of pleiotropy. If the distributions of the variant(s)-trait 1 effects and variant(s)-trait 2 effects are independent, the effect estimate obtained from Egger regression is equivalent to the MR estimate obtained from inverse variance weighted analysis and can thus be used to infer causality between trait 1 and 2. Recently, Bowden and colleagues proposed a summary data-based step-wise analysis framework which applies all three of the above methods to differentiate between the scenarios of no pleiotropy, pleiotropy without heterogeneity and pleiotropy with heterogeneity [146]. By applying this framework to summary data from two GWAS the authors showed that the observed association between plasma urate levels and cardiovascular disease was likely to be due to pleiotropy rather than a causal link, as evident from heterogeneity in the MR estimates from the 31 variants analysed.

### 2.9. Detecting spurious pleiotropy

In addition to methods for detecting cross-phenotype effects, a recently published tool (BUHMBOX) aims to distinguish heterogeneity in disease cases from true pleiotropy between two diseases [149]. It compares risk allele frequency of variants associated with one disease (D1) in the cases of a second disease (D2). By deriving a test statistic from the correlation matrix between all risk loci, BUHMBOX tests whether the D1 risk alleles are enriched in a subgroup of D2 cases (high correlation) or whether they are evenly distributed across all D2 cases (low correlation). The former yields a significant test statistic and is indicative of heterogeneity. On the other hand, a non-significant statistic could be the result of either true pleiotropy or insufficient power.

To our knowledge, BUHMBOX is currently the only method aimed at identifying spurious pleiotropy. BUHMBOX is not agnostic (i.e. D1 and D2 need to be specified by the user) and it is only applicable to disease traits.

## 3. Future directions

Pleiotropy will continue to grow as a key research area of human genetics as data availability increases.

A key question that has been the subject of debate in evolutionary biology [6] and is likely to receive more attention in human genetics is how to define a trait. For example, if two different anthropometric traits always covary, is it accurate to treat them as separate traits or are they actually two measurements of the same underlying biological phenotype? Such questions will be especially important in multivariate models where the inclusion of highly correlated traits can be costly in terms of power.

A further consideration is whether functional measurements, such as gene or protein expression, constitute traits in themselves or simply intermediate steps between genes and phenotype. This is especially important when attempting to draw biological conclusions from multi-trait analyses. MR approaches will be necessary to correctly infer causality, and indeed some work has already been done with regard to this question [150].

It is increasingly common for researchers to be confronted with sample numbers in the hundreds of thousands, and an important next step will be to develop fast and efficient algorithms with good scalability. In contrast to single-trait GWAS, where analysis approaches are now fairly standardized, multi-trait methods vary considerably in the statistical approaches they employ. Ensuring comparability and replicability of results will be a key challenge in pleiotropy research moving forward.

As data on cross-phenotype associations accumulate and our understanding of molecular links between diseases grows, these insights will be invaluable for drug development and repurposing, and for personalized medicine.

## Data accessibility

This article has no additional data.
